# Psychometry

**Published:** 1887-07

**Authors:** 


### HALL'S
### JOURNAL OF HEALTH

TRUTH DEMANDS NO SACRIFICE; ERROR CAN MAKE NONE.

VOL. 34. JULY, 1887 No 7.

### PSYCHOMETRY.

It was in the autumn of 1842, that Professor J. Rodes Buchanan discovered and proved by a series of experiments, the existence of wonderful psychological powers in man, which had never before been recognized. "In that single discovery," says Dr. Buchanan in an article published seven years later, "lay the germ of a science of lofty pretentions, and so wonderful in its facts as to be difficult of belief, if not utterly incredible, to the greater portion of our scientific men."

Dr. Buchanan coined a name for the embryo science out of two Greek words, which signify *soul measuring*. Among those persons who were early invited to witness exhibitions illustrative of psychometry, was the eminent divine, poet and scholar, John Pierpont, who subsequently introtroduced it to public notice in a poem delivered at the celebration of the one hundred and fiftieth year of the alumni of Yale College, in the course of which, after some highly complimentary allusions to its discoverer, the poem proceeds :

"Mysterious science ! that has now displayed
How fearfully and wonderfully made"
Is man, that even his touch can catch the mind,
That long has left material things behind !
* * * * * * * *
The very page that I am tracing now,
With tardy fingers and a careworn brow,
To other brows by other fingers press'd,
Shall tell the world, not what I had been deem'd,
Nor what I passed for, nor what I had seem'd,
But what I *was !* Believe it, friends, or not,
To this high point of progress have we got,
We stamp ourselves on every page we write ;—
Send you a note to China or the pole—
Where 'er the wind blows or the waters roll—
That note conveys the measure of the soul !"

In his introduction to his late work on Psychometry, after a course of investigation running through some fifteen years, with the most ample opportunities of inquiry and experiment, Dr. Buchanan says: "As a science and philosophy, psychometry shows the nature, the scope and the *modus operandi* of those divine powers in man, and the anatomical mechanism through which they are manifested : while as an art, it shows the method of utilizing these psychic faculties in the investigation of character, disease," etc. etc.

It is one of the most startling propositions which the human mind is capable of grasping, that the delicately organized sensitive, upon being brought into physical contact with any substance, however slight, is able to perceive not only its nature and the uses to which it has been subjected, but by the same inexplicable, nay, incomprehensible, means, the impress which has been left upon it by the brain that planned or the hand that fashioned it, even though linked to the crumbling runis of a pre-historic age; what is more and yet more wonderful, the *descriptio personne* and personal traits as well, of those same individuals.

Through all these years where has the subtle force resided, how maintained itself, which awakes at a fingers touch, and flashing along the sensory nerves to the brain, communicates with the clearness and intelligence of a living person, that wonderful tale of the past, which it is ever ready to reveal, but never surrenders ?

It is said that the beginning of wisdom is a realizing sense of how little we really know, and it would seem that here is a vast and hitherto unexplored field, the entrance to which has been laid bare by the untiring persistency of its discoverer.

Many individuals possess this wonderful psychological gift, many indeed, who are unconscious of it, and others, who have been led by its proper recognition, to encourage and cultivate it to a degree of exquisite fineness, susceptibility and consequent power, and numerous are the instances of accurate readings and marvelous predictions made through these instrumentalities, some of which, being distasteful, were rejected at the time with rudeness and incredulity, but were subsequently verified in a manner quite extraordinary.

The keys by means of which such information is arrived at are various —a pebble, a mineral fragment, a scrap of writing, a lock of hair, indeed, almost anything may serve. For example, we have in our possession a fragment of one of the Pyramids, which, upon being placed in the hands of a psychometist, as this class of sensitives are called, who had no knowledge of its history, an accurate description of the great pile was given with a great deal of other information, which can only be verified by future discoveries.

As applied to medicine and surgery, the information derived from these sources is, oftentimes, of very great value. But few, indeed, of the old school practitioners have been found willing to accept anything so far in advance of the rules laid down in their text books. Upon this particular subject Dr. Buchanan says: "I have had no hesitation in relying upon a psychometric diagnosis by Mrs. B., and directing the treatment of patients whom I had never seen, but whose assurances of correct description and satisfactory cures have been all I could expect." * * * "In no case have patients failed to recognize the truth of the diagnosis." This is saying much for the new science as an agent in the diagnosis and cure of disease.

But, although Dr. Buchanan was the first to proclaim the existence of that faculty of the brain, which renders these things possible through cultivation and constant use, and to it give it a significant name, and accord it place second to no other, in the realm of natural science, it is evident that the faculty itself is far from being a new development, nor was its exercise unknown and unrecognized, as one of the mysteries which gave prominence to those earlier manifestations of invisible forces directed by intelligence, with which, in later years, the world has become far better acquainted, through the efforts of such eminent investigators as Professors Buchanan, Britton, Hare, Denton, Epes, Sargant, and a host of others, sufficiently independent of established methods and beliefs, to declare their honest convictions.

The last named author devoted his later years almost exclusively to the study of the occult forces and their relation to human affairs.

It was an axiom with him that "*Memory is imperishable*," that all thought and all actions leave their eternal record in the organic structure of our very souls. Nothing happens, not the most fleating and trivial occurence of our lives that may not be ages and œons hence, reproduced to our own consciousness, as well as to that of others, independently of our own will or co-operation."

"There is," says Voltaire, "a power that acts within us without consulting us," and both Coleridge and Abercrombie mention the case of an ignorant young woman, who during a fever, talked incessantly in Latin, Greek and Hebrew, without actually knowing a word of either language, but it was ascertained that she had lived with a learned man who was a proficient linguist.

"This authenticated case" says Coleridge, "furnishes us both proof and instance that reliques of sensation may exist for an indefinite time, in a latent state, in the very same order in which they were originally impressed."

It was the genius of Franklin that discovered the law of those electric forces, which had existed for all time, and set a guard to their destroying touch, till now, they are produced at will, and governed by the hand of man. We should never be too ready to condemn a thing, because we have not brought ourselves to understand it, for many a truth lies below the surface, and must be diligently sought after, and he who refuses to make acquaintance with it, when discovered and brought to light, out of the prejudices of custom or education, is about as wise as a blind man would be to refuse the offer of sight, lest it might destroy the images he had formed in his mind of objects which bordered the path along which he was accustomed to grope his uncertain way.

"'Tis immortality deciphers man,
And opens all the mysteries of his make.
Without it, half his instincts are a riddle;
Without it, all his virtues are a dream."

